# Comparative Study of Cellulose Nanocrystals from Young and Mature Coconut Husks as Reinforcement Agents in Sustainable Gelatin-Based Films

**DOI:** 10.3390/polym18060708

**Published:** 2026-03-14

**Authors:** Pimonpan Kaewprachu, Warinporn Klunklin, Chalalai Jaisan, Saroat Rawdkuen, Papungkorn Sangsawad, Wirongrong Tongdeesoontorn, Passakorn Kingwascharapong, Supaluck Kraithong

**Affiliations:** 1 Faculty of Agro-Industry, Chiang Mai University, Samut Sakhon 74000, Thailand; chalalai.jai@cmu.ac.th; 2 Division of Marine Product Technology, Faculty of Agro-Industry, Chiang Mai University, Chiang Mai 50100, Thailand; warinporn.k@cmu.ac.th; 3 Unit of Innovative Food Packaging and Biomaterials, School of Agro-Industry, Mae Fah Luang University, Chiang Rai 57100, Thailand; saroat@mfu.ac.th (S.R.); wirongrong.ton@mfu.ac.th (W.T.); 4 School of Agro-Industry, Mae Fah Luang University, Chiang Rai 57100, Thailand; 5 Postharvest Technology and Innovation in Animal Unit, Institute of Agricultural Technology, Suranaree University of Technology, Nakhon Ratchasima 30000, Thailand; papungkorn@sut.ac.th; 6 School of Animal Technology and Innovation, Institute of Agricultural Technology, Suranaree University of Technology, Nakhon Ratchasima 30000, Thailand; 7 Department of Fishery Products, Faculty of Fisheries, Kasetsart University, Bangkok 10900, Thailand; passakorn.ki@ku.th; 8 Guangxi Key Laboratory of Marine Drugs, Institute of Marine Drugs, Guangxi University of Chinese Medicine, Nanning 530200, China; supak0504@gmail.com

**Keywords:** agricultural waste, biopolymer nanocomposite, circular economy, coconut husk, cellulose nanocrystals, gelatin films, sustainable packaging, waste valorization

## Abstract

Cellulose nanocrystals (CNCs) are highly desirable nanomaterials for reinforcing biopolymer films. Coconut husks are generated in massive quantities after harvesting and processing, leading to waste management issues. This study isolated and characterized CNCs from young (y-CNCs) and mature (m-CNCs) coconut husks via acid hydrolysis (32% H_2_SO_4_, 50 °C, 5 h), comparing them with commercial CNCs (c-CNCs) to evaluate their performance in gelatin-based films. TEM confirmed rod-shaped morphology for all CNCs. Notably, m-CNCs exhibited a smaller particle size (199 nm), a higher surface charge (−46.8 mV), and superior crystallinity (63.98%), demonstrating properties comparable to c-CNCs. FTIR and XRD confirmed characteristic cellulose functional groups and crystalline structure, while TGA demonstrated excellent thermal stability above 300 °C for all samples. Incorporation of CNCs into gelatin films significantly improved tensile strength (from 15.63 to 24.93 MPa) and reduced water vapor permeability (from 2.65 to 2.43 × 10^−10^ g m m^−2^ s^−1^ Pa^−1^; *p* < 0.05). These findings demonstrate how coconut husk residues can be upcycled into high-value nanomaterials fostering economic growth with innovation in sustainable manufacturing. This research also promotes responsible waste utilization, highlighting the benefits of biodegradability and a reduced carbon footprint for sustainable food packaging applications.

## 1. Introduction

Petroleum-based polymers are non-biodegradable, and their manufacturing, recycling, and disposal processes release harmful pollutants into the environment. Global plastic production reached 430.9 million tonnes in 2024, with fossil-based plastics still accounting for 89.5% of total output, while packaging applications represent approximately 40% of total demand [1]. Despite growing environmental awareness, only a small fraction of plastic waste is effectively recycled, with the vast majority accumulating in landfills or leaking into natural environments [2]. The global escalation of plastic pollution has driven urgent research into sustainable alternatives, particularly for food packaging applications. Consequently, biopolymers derived from sustainable sources have emerged as a viable approach to address these environmental challenges. The increasing global demand for sustainable development and efficient waste utilization has intensified research into high-value applications for lignocellulosic agricultural by-products.

The coastal regions and islands of Asia and Oceania, particularly the Philippines, Indonesia, and Thailand, account for approximately 60% of the global coconut cultivation area, which totals around 11.2 million hectares [3]. With a global production of approximately 62–65 million tonnes annually, these three countries collectively generate substantial quantities of lignocellulosic by-products, corresponding to an estimated 11–13 million tonnes of coconut husk biomass and over 4 million tonnes of shell residues each year. In this context, the coconut (*Cocos nucifera* L.) industry generates substantial quantities of residues. In Samut Sakhon, Thailand, both young and mature coconut husks are primary agricultural wastes; young husks are typically generated from the fresh fruit market (coconut water), while mature husks are by-products of the coconut milk industry. After harvesting and processing coconuts, the husk accounts for approximately 60–70% of the total coconut weight [4]. The amount of coconut husk waste produced continues to grow, and inadequate management of these materials poses significant environmental risks. However, coconut husks represent a major source of cellulose with significant potential as a starting material for cellulose and nanocellulose production. Therefore, the valorization of these by-products could simultaneously mitigate agricultural waste problems and provide a pathway to generate high-value-added products.

Nanocellulose can be prepared from cellulose by disrupting its native amorphous regions via chemical, mechanical, or biological methods or by combining these approaches, yielding materials less than 100 nm in diameter and several micrometers in length [5]. Among the various forms of nanocellulose, cellulose nanocrystals (CNCs), also known as nanocrystalline cellulose, nanocrystals of cellulose, or cellulose nanowhiskers, are short and rod-like or whisker-shaped, with a typical diameter of 2–20 nm and a length of 100–500 nm [5]. They are usually obtained through acid hydrolysis that selectively removes amorphous regions while preserving highly ordered crystalline domains [6]. CNCs have received considerable attention owing to their remarkable attributes, such as elevated crystallinity, an extensive specific surface area, a high aspect ratio, superior mechanical strength, thermal stability, biocompatibility, and biodegradability [7,8]. These characteristics position CNCs as reinforcing agents for biopolymer composites. The crystallinity index (CrI) and particle size of CNCs significantly influence their reinforcement efficiency, with higher crystallinity and optimal aspect ratios correlating with superior mechanical performance in composite applications [9].

Lignocellulosic agricultural waste represents an abundant, renewable, and economically viable source for CNC production. Coconut husk, a by-product of the coconut industry that is often discarded or underutilized, is particularly promising due to its fibrous structure rich in cellulose, hemicellulose, and lignin [4,10]. This makes it a readily available and cost-effective raw material for extracting cellulose and its nanostructured derivatives. However, the chemical composition and structural integrity of coconut husks vary significantly depending on the stage of maturity. Coconut husk exists in two distinct developmental phases: the softer, less-lignified young (green) husk and the highly lignified, mechanically stronger mature (brown) husk. Young and mature coconut husks differ markedly in their cellulose-to-lignin ratios, with untreated husks typically containing approximately 30–41% lignin, 21–35% cellulose, and 13–17% hemicellulose, although these proportions shift during fruit maturation [4]. Mature husks often undergo extensive lignification, which may alter the accessibility of cellulose to chemical treatments and subsequently affect the crystallinity, morphology, and thermal stability of the isolated CNCs compared to their younger counterparts.

Gelatin, derived from collagen, has gained significant attention as a biopolymer for packaging applications due to its biodegradability, biocompatibility, film-forming ability, and non-toxicity [11]. However, neat gelatin films often suffer from poor mechanical strength and water resistance, which limit their practical applications. To overcome these drawbacks, the incorporation of nanofillers, specifically CNCs, has emerged as an effective strategy to enhance the properties of gelatin-based films. While CNCs have demonstrated promise as reinforcing agents, there is limited information on the effect of source material maturity on the characteristics and reinforcement performance of CNCs isolated from coconut husks. Coconut husk-derived CNCs could improve film properties and expand the range of sustainable packaging applications. Therefore, this study aimed to isolate and characterize CNCs derived from young and mature coconut husks—denoted as y-CNCs and m-CNCs, respectively—via sulfuric acid hydrolysis, and to evaluate their effectiveness as reinforcing agents in gelatin-based films. The morphological, physicochemical, and thermal characteristics of the y-CNCs and m-CNCs were compared with c-CNCs. The reinforcement performance of these CNCs in gelatin-based films was assessed through mechanical and barrier property measurements. This research provides a pathway for transforming agricultural waste into high-performance nanomaterials while identifying optimal biomass sources for sustainable food packaging development.

## 2. Materials and Methods

### 2.1. Materials

Young and mature coconut husks were obtained from a local coconut factory (Ban Phaeo, Samut Sakhon, Thailand). Glacial acetic acid, sodium hydroxide (NaOH), hydrogen peroxide (H_2_O_2_), and sulfuric acid (H_2_SO_4_) were purchased from RCI Labscan Ltd. (Bangkok, Thailand). c-CNCs were purchased from Nanografi Co., Ltd. (Ankara, Turkey). These nanocrystals are 10–20 nm in diameter and 300–900 nm in length. Bovine gelatin (250 Bloom) was obtained from Thai Food and Chemical Co., Ltd. (Bangkok, Thailand). All materials and chemicals used in our study were of analytical grade.

### 2.2. Preparation of Cellulose from Coconut Husk

Young and mature coconut husks were collected and processed for cellulose extraction. Following exocarp (outer skin) removal, the mesocarp was sectioned into small fragments, washed thoroughly with distilled water, and oven-dried at 70 °C for 24 h. The dried material was pulverized using a high-speed grinder and passed through a 60-mesh sieve prior to extraction. Cellulose was isolated according to the method described by Klunklin et al. [10]. The powdered material underwent alkaline treatment using a 15% (*w*/*v*) NaOH solution at a solid-to-liquid ratio of 1:15, maintained at 90 °C for 3 h with continuous agitation (IKA RW 20 digital, BEC Thai Bangkok Equipment & Chemical Co., Ltd., Bangkok, Thailand) at 100 rpm. The resulting slurry was filtered and washed with water until a neutral pH was reached. The residue was subsequently dried in an oven at 60 °C for 24 h to yield cellulosic pulp. The pulp underwent a multi-step bleaching process: initial bleaching with 10% (*v*/*v*) H_2_O_2_ solution (1:20 ratio) at 70 °C for 2 h, followed by two sequential treatments with 1.4% (*w*/*v*) sodium chlorite (NaClO_2_) solution (pH adjusted to 4.0 using 5% acetic acid) at a 1:30 ratio and 80 °C for 5 h each. After each bleaching stage, the material was filtered and rinsed with water until a neutral pH was reached. The purified cellulose fibers from young and mature coconut husks (y-cellulose and m-cellulose, respectively) were oven-dried at 60 °C for 24 h and stored in sealed plastic bags at ambient temperature until further use.

### 2.3. Extraction of CNCs from Y-Cellulose and M-Cellulose

CNCs were isolated following a modified form of a published protocol [12]. Dried cellulose (5 g) was subjected to acid hydrolysis using 150 mL of 32% (*v*/*v*) sulfuric acid at 50 °C for 5 h. The reaction was terminated by adding 150 mL of cold distilled water, followed by centrifugation (1248R, Labogene, Lynge, Denmark) at 8000 rpm for 10 min to separate the hydrolyzed material. The supernatant was collected and neutralized to pH 7.0 using 5% (*w*/*v*) NaOH solution. The neutralized suspension was then homogenized at 12,000 rpm for 15 min using an IKA T25 digital ULTRA-TURRAX homogenizer (IKA®-Werke GmbH & Co. KG, Staufen, Germany). Subsequently, the CNC suspension was sonicated in an ultrasonic bath (ice bath; GT sonic D20, Shenzhen, China) at 40 kHz for 30 min. Following ultrasonication, the suspension was centrifuged at 6000 rpm for 10 min, with the supernatant collected and the process was repeated until no precipitate was observed. The CNC suspension was sonicated again in an ultrasonic bath (ice bath) for 30 min. Finally, the suspended CNCs were stored in a freezer at −20 °C and subsequently freeze-dried in a GFD-3H freeze-dryer (Grisrianthong, Samut Sakhon, Thailand).

### 2.4. Characterization of CNCs from Coconut Husks

#### 2.4.1. Morphology

Field emission scanning electron microscopy (FESEM, JSM-IT800SHL, JEOL, Tokyo, Japan) was used to analyze the morphology of CNCs. Observations were carried out at a voltage of 20 kV and 50,000× magnification. CNCs were dispersed in distilled water to a concentration of 0.25% (*w*/*v*) and sonicated for 30 min.

Transmission electron microscopy (TEM) was used for morphology-based measurements. The TEM image of the obtained CNC suspension (0.3% *w*/*v*) was observed on a Talos F200X G2 transmission electron microscope (Thermo Fisher Scientific, Waltham, MA, USA). The sample was negatively stained with 1% aqueous uranyl acetate for 5 min. After removal of the excess solution, the sample was dried at room temperature prior to TEM. The ImageJ software (version 1.53k) (National Institutes of Health, Bethesda, MD, USA) was used to measure the length and width of the CNCs. The diameter and length of at least 100 randomly selected individual nanocrystals were measured from multiple TEM images for each sample. The aspect ratio was calculated as the ratio of length to diameter. The results are reported as the mean ± standard deviation (SD).

#### 2.4.2. Functional Group Analysis

Fourier-transform infrared (FTIR) spectroscopy was employed to characterize the functional groups present in cellulose and coconut husk-derived CNCs using a Spectrum GX instrument (Perkin Elmer, Waltham, MA, USA). Approximately 2 mg of each sample was finely ground, mixed with potassium bromide (KBr), and compressed into pellets for analysis. Spectral measurements were performed in transmission mode over the wavenumber range of 400–4000 cm^−1^ with a spectral resolution of 4 cm^−1^, averaging 64 scans per sample.

#### 2.4.3. X-Ray Diffraction (XRD) Analysis

The XRD patterns were obtained using an X-ray diffractometer (D8 Discover, Bruker AXS, Karlsruhe, Germany) with Ni-filtered Cu Kα radiation at 40 kV and 40 mA. The diffraction data were collected from 2θ = 5–50°. The CrI was estimated using the XRD peak height method and calculated using Equation (1) [13]:

CrI = [(Imax − Iam)/Imax] × 100%(1)

where Iam is the intensity of amorphous peak cellulose at 2θ = 18° and Imax is the highest crystalline peak of the maximum intensity at 2θ = ~22°.

#### 2.4.4. Particle Size Analysis

The particle size distribution of colloidal CNC suspensions was measured using dynamic light scattering (DLS, Zetasizer Nano ZSP, Malvern, UK) in the range of 0.6–6000 nm. The CNC suspensions were prepared at a concentration of 0.02% with sonication for 5 min (pulses of 2 s on and 1 s off) in an ice bath using an ultrasonic processor (Cole-Parmer®, 130-Watt, 20 kHz, Vernon Hills, IL, USA) to improve sample dispersibility and to reduce agglomeration. The DLS measurements were performed at room temperature (25 °C).

#### 2.4.5. Zeta Potential

The colloidal stability and particle size of CNCs were evaluated using zeta potential and DLS measurements (Zetasizer Nano ZSP) based on electrophoretic light scattering principles. All measurements were performed on CNC suspensions at a concentration of 0.02% (*w*/*v*) and maintained at 25 °C.

#### 2.4.6. Thermal Properties

Thermal stability of cellulose and CNCs was analyzed using thermogravimetric analysis (TGA; TG 209 F3, NETZSCH, Tarsus, Germany). The dried samples (5–10 mg) were heated from 25 to 500 °C at a heating rate of 10 °C/min under a nitrogen ambient of 20 mL/min.

### 2.5. Preparation of Gelatin-Based Films Reinforced with CNCs

The concentrations of gelatin and CNCs were each set at 3% (*w*/*w*, based on protein content), as determined from preliminary studies. Gelatin films were prepared following the method described by Kaewprachu et al. [14]. Briefly, gelatin powder (3% *w*/*w*, based on the protein content) was dissolved in distilled water and heated at 60 °C for 30 min. The CNC suspension, previously sonicated for 5 min to ensure uniform dispersion, was then incorporated into the gelatin film-forming solution (FFS) at a final concentration of 3% (*w*/*w*, based on the protein content) and stirred for 15 min. Subsequently, glycerol (30% *w*/*w*, based on the protein content) was added as a plasticizer to the mixture and continuously stirred for an additional 10 min to ensure homogeneity. The FFS (5 ± 0.01 g) was cast onto a rimmed silicone resin plate (50 × 50 mm) and placed in an oven at 35 °C overnight and dried in a ventilated oven environmental chamber at 50% ± 5% relative humidity (RH) and 25 ± 0.5 °C for 24 h. Finally, the films were peeled off from the plates and denoted as G (neat gelatin film), G/y-CNC (gelatin film reinforced with y-CNCs), G/m-CNC (gelatin film reinforced with m-CNCs), and G/c-CNC (gelatin film reinforced with c-CNCs).

### 2.6. Characterization of the Gelatin-Based Films Reinforced with Nanocellulose

#### 2.6.1. Scanning Electron Microscope (SEM)

The surface and the cross-section of the gelatin-based films reinforced with nanocellulose was examined using SEM (JSM-IT700HR, JEOL). The SEM images were captured at 500× magnification for the surface and at 1000× magnification for the cross-section with an acceleration voltage of 10 kV.

#### 2.6.2. FTIR Spectroscopy

The functional groups of the gelatin-based films reinforced with nanocellulose were analyzed using an FTIR spectrometer (Spectrum GX, Perkin Elmer) in the range of 4000 to 400 cm^−1^ with a resolution of 4 cm^−1^ and 64 scans.

#### 2.6.3. Film Thickness

Film thickness was determined using a digital micrometer (Mitsutoyo Co., Ltd., Kanagawa, Japan) with a measurement precision of 0.001 mm. For each sample, thickness was measured at six different locations across ten replicate films.

#### 2.6.4. Mechanical Properties

Tensile strength (TS) and elongation at break (EAB) of gelatin-based nanocomposite films were determined using a texture analyzer (TA.XT plus, Stable Micro Systems, Godalming, UK) equipped with a 50 kg load cell. Film specimens were cut into rectangular strips (2 × 5 cm^2^), and five replicates per sample were tested at a crosshead speed of 1.5 mm s^−1^ with an initial grip separation of 30 mm. The testing procedures followed ASTM standard method D882-97 [15].

#### 2.6.5. Water Vapor Permeability (WVP)

WVP of nanocellulose-reinforced gelatin films was measured according to a modification of the ASTM E96-95 method [16]. Film specimens were mounted and sealed over aluminum permeation cups (with a 70 mm diameter opening) containing 20 g of anhydrous silica gel as desiccant to maintain approximately 0% RH inside the cup. The sealed assemblies were placed in a climate chamber (KMF 115, Binder GmbH, Tuttlingen, Germany) maintained at 25 °C and 75% RH for 8 h. The cup weight was recorded at 1 h intervals to determine the water vapor transmission rate. WVP is expressed as g m m^−2^ s^−1^ Pa^−1^. Three replicate measurements were performed for each film formulation.

#### 2.6.6. Water Contact Angle (WCA)

WCA measurements were performed using an OCA-20 contact angle microscope (DataPhysics, Filderstadt, Germany) to assess the surface wettability of nanocellulose-reinforced gelatin films. A 5 µL droplet of distilled water was deposited onto the film surface, and the contact angle was measured immediately. Magnified images of the water droplet were captured to determine the WCA.

#### 2.6.7. Ultraviolet–Visible Light (UV–Vis) Barrier Properties

The UV–VIS barrier properties of the gelatin-based films with and without nanocellulose were determined at wavelengths ranging from 200 to 800 nm with a UV–Vis spectrophotometer (G105 UV-VIS, Thermo Scientific Inc., Waltham, MA, USA).

### 2.7. Statistical Analysis

Statistical analysis was performed using SPSS software (SPSS for Windows version 16.0, SPSS Inc., Chicago, IL, USA). The data were subjected to an analysis of variance (ANOVA), and significant differences between means were identified using Duncan’s multiple range test at a significance level of *p* < 0.05.

## 3. Results and Discussion

### 3.1. Appearance and Percentage of Yield

**Figure 1:**
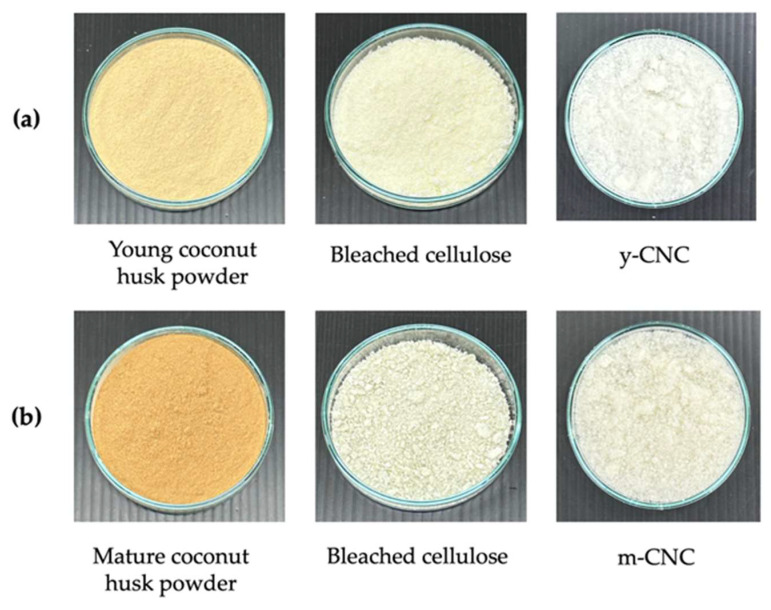
Appearance of cellulose and cellulose nanocrystals (CNCs) from young coconut husk (y-CNC) (**a**) and mature coconut husk (**b**) (m-CNC).

The appearance of the coconut husk powder, cellulose, and CNCs isolated from young and mature coconut husk is shown in Figure 1. The young coconut husk had a warm yellow color, while the mature coconut husk was golden brown. This color difference can be attributed to variations in the lignin content associated with biomass maturity [10]. After the bleaching step, the bleached cellulose appeared light yellow for young coconut husk and off-white for mature coconut husk, indicating the formation of almost pure cellulosic material. These observed color changes confirmed the effective elimination of non-cellulosic substances, including lignin, hemicelluloses, pectin, and other contaminants from the coconut husk fiber structure. Following acid hydrolysis and freeze-drying, y-CNCs and m-CNCs appeared as a white powder, demonstrating successful isolation and purification.

The m-CNC yield (29.64%) was significantly higher than the y-CNC yield (20.59%). This difference can be attributed to maturity-related increases in the cellulose content and crystallinity associated with secondary cell wall development [17,18]. During plant maturation, the deposition of cellulose in secondary cell walls increases both the total cellulose content and the proportion of highly ordered crystalline regions [19,20]. Consequently, the higher proportion of stable crystalline domains in mature coconut husk results in more recoverable CNCs following selective acid hydrolysis of amorphous regions, as the crystalline cellulose resists acid degradation and is preferentially retained as nanocrystals.

### 3.2. Morphology of CNCs

**Figure 2:**
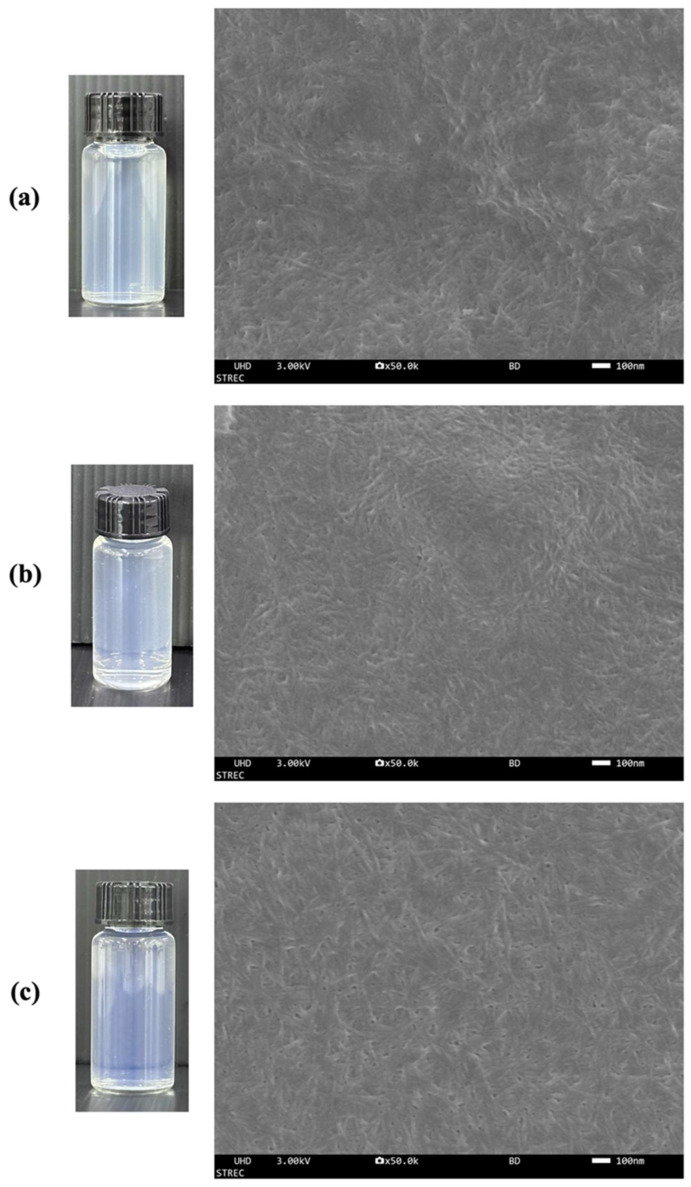
FESEM of cellulose nanocrystals from young coconut husk (**a**) and mature coconut husk (**b**) in comparison with commercial nanocellulose (**c**). Magnification: 50,000×. Scale bar: 100 nm.

The photographs of suspensions (0.1% *w*/*v*) of the y-CNCs and m-CNCs, compared to the c-CNCs, are presented in Figure 2. All CNC suspensions exhibited transparency with slight bluish opalescence and remained stable without visible sedimentation, indicating excellent colloidal stability at low concentrations. This behavior is attributed to negatively charged sulfate ester groups grafted onto the CNC surface during sulfuric acid hydrolysis, which promote electrostatic repulsion between particles and prevent aggregation [21].

Figure 2 presents FESEM images of the y-CNCs and m-CNCs in comparison to the c-CNCs. All CNC samples exhibited the characteristic rod-like morphology typical of CNCs, confirming successful isolation from both young and mature coconut husks. In the dry state, the CNCs formed interconnected, web-like network structures due to strong hydrogen bonding interactions between individual particles. This three-dimensional network architecture is commonly observed in nanocellulose materials and is attributed to the high density of surface hydroxyl groups that facilitate inter-particle bonding upon solvent evaporation [8]. Visual comparison of the FESEM images revealed subtle morphological differences among the samples. The y-CNCs displayed a more entangled and loosely aggregated network structure (Figure 2a), while the m-CNCs and c-CNCs exhibited more compact and uniform network arrangements (Figure 2b,c). These observations suggest differences in particle size distribution and inter-particle interactions that may be influenced by biomass maturity and processing conditions. Similar rod-like morphologies and network-forming behavior have been reported for CNCs derived from various biomass resources, including *Cucumis sativus* peels, rice straw, poplar wood, and commercial CNCs [22,23,24], confirming that the isolated materials are consistent with CNC characteristics reported in the literature.

**Figure 3:**
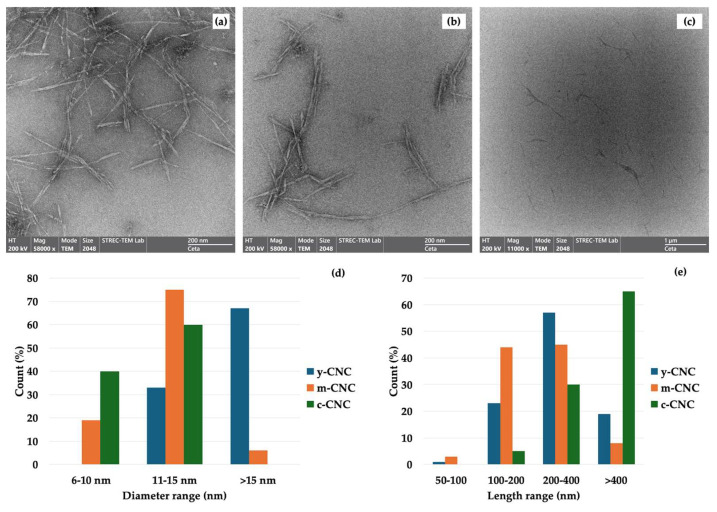
TEM of cellulose nanocrystals (CNCs) from young coconut husk (**a**), mature coconut husk (**b**), commercial CNC (**c**), and diameter (**d**) and length contribution (**e**). Magnification: 58,000× for coconut husk and 11,000× for commercial CNC. Scale bar: 200 nm for y-CNC and m-CNC; Scale bar: 1 µm for commercial CNC.

TEM was used to further assess CNC morphology (Figure 3). All samples exhibited the characteristic rod-like or needle-like morphology of CNCs, confirming the effective removal of non-cellulosic components and successful isolation of CNCs through acid hydrolysis [6,19]. This morphology is consistent with that reported in the literature for coconut-derived materials and other lignocellulosic sources. Similar rod-like and needle-like structures have been extensively documented: Zhang et al. [21] reported that CNCs from bleached pulp board exhibited a “narrow, two-pointed” rod-like structure. CNCs from corn stalk displayed needle-like morphology [25]. Beyene et al. [26] observed that CNCs from filter paper and wood pulp fibers presented a rod/needle shape structure with unequal lengths and widths. Dai et al. [27] reported needle-shaped CNCs from wood pulp. Finally, Ichwan et al. [28] observed whisker-like morphologies in CNCs from empty oil palm fruit bunches. These consistent observations across diverse biomass sources confirm that the acid hydrolysis process selectively degrades amorphous regions while preserving the crystalline structure, resulting in the characteristic rod-shaped nanocrystals.

However, there were notable morphological distinctions among the CNCs. The y-CNCs (Figure 3a) displayed longer rods with a higher degree of entanglement and loose aggregation, forming interconnected network structures. This elongated morphology and tendency to aggregate can be attributed to the less compact cell wall architecture and higher proportion of amorphous cellulose regions in young biomass, which reduce the efficiency of acid penetration and limit the extent of hydrolytic cleavage during sulfuric acid hydrolysis [6,29]. Consequently, partial fragmentation of cellulose microfibrils occurs, resulting in longer nanocrystals that are more prone to inter-particle interactions through hydrogen bonding and physical entanglement.

In contrast, the m-CNCs (Figure 3b) exhibited shorter nanocellulose rods with reduced aggregation and improved dispersion compared to the y-CNCs, although the particles were completely separated. While there was still some bundling and particle overlap, the m-CNCs showed less extensive entanglement than the y-CNCs, consistent with more efficient hydrolysis of mature coconut husk. This morphology reflects more effective and selective hydrolysis of amorphous domains, facilitated by the increased secondary cell wall development, higher structural organization, and greater cellulose packing density characteristic of mature lignocellulosic fibers [17,19,20]. Such structural maturity enhances the susceptibility of non-crystalline regions to acid attack while preserving crystalline domains, leading to the formation of shorter and more uniform CNCs [18]. The reduced degree of entanglement and improved particle separation observed in the m-CNCs are therefore consistent with a more homogeneous nanocellulose population at the nanoscale.

The c-CNCs (Figure 3c) displayed the most uniform morphology with well-dispersed, individual rod-like structures showing minimal aggregation. The nanocrystals exhibited consistent dimensions and excellent separation, reflecting optimized and standardized industrial hydrolysis conditions typically employed for wood-based cellulose sources [9,19]. The superior dispersibility of the c-CNCs can be attributed to controlled processing parameters that ensure complete removal of amorphous regions while maintaining uniform crystal dimensions. Overall, the TEM observations demonstrated that biomass maturity plays a critical role in governing nanocellulose morphology by controlling the extent of fibril fragmentation, aggregation behavior, and dimensional uniformity during acid hydrolysis. Similar maturity-dependent trends in CNC dimensions and dispersion characteristics have been reported for other lignocellulosic sources, including banana peel and wood-based materials, further supporting the influence of cell wall architecture on nanocellulose structure [18,29]. The superior morphological characteristics of the m-CNCs, particularly their reduced aggregation and improved uniformity compared to the y-CNCs, suggest enhanced potential as a reinforcing agent in biopolymer composite applications.

Quantitative TEM image analysis of 100 randomly selected particles per sample revealed systematic differences in CNC dimensions among the three sources (Figure 3d,e). Diameter distribution analysis (Figure 3d) showed that the c-CNCs exhibited the narrowest distribution (3–16 nm), with 40% of particles in the 6–10 nm range and 60% in the 11–15 nm range, yielding a mean diameter of 11 ± 2 nm. The m-CNCs showed an intermediate distribution (4–19 nm) with 75% of particles in the 11–15 nm range and a mean diameter of 13 ± 3 nm. In contrast, the y-CNC displayed the largest and broadest distribution (11–20 nm), with only 33% of particles in the 11–15 nm range and the majority (67%) exceeding 15 nm, yielding a mean diameter of 16 ± 4 nm.

Length distribution analysis (Figure 3e) revealed more pronounced differences. The y-CNCs exhibited a broad distribution (50 to >400 nm) with 57% of particles in the 200–400 nm range, 23% in the 100–200 nm range, and 19% exceeding 400 nm (mean ± SD: 280 ± 70 nm). The m-CNCs showed a more balanced distribution, with 44% in the 100–200 nm range, 45% in the 200–400 nm range, and only 8% exceeding 400 nm (mean ± SD: 220 ± 55 nm). The c-CNCs displayed distinctly longer particles, with 60% exceeding 400 nm and 37% in the 200–400 nm range (mean ± SD: 450 ± 95 nm).

The calculated aspect ratio was 18 ± 5 for the y-CNCs, 17 ± 4 for the m-CNCs, and 41 ± 9 for the c-CNCs, falling within the typical range (10–70) reported for CNCs from lignocellulosic sources [30]. Notably, the y-CNCs and m-CNCs exhibited nearly identical aspect ratios despite differences in their dimensional profiles. The y-CNCs possessed a larger diameter (16 nm) with longer average length (280 nm), while the m-CNCs had a smaller diameter (13 nm) and a shorter length (220 nm), resulting in similar aspect ratios. However, the m-CNCs demonstrated superior dimensional uniformity, with narrower size distributions for both diameter and length compared to the broader, more heterogeneous distributions of the y-CNCs. The progressive decrease in the mean diameter from y-CNCs (16 nm) to m-CNCs (13 nm) to c-CNCs (11 nm), coupled with the increasingly narrow size distributions, indicates that both coconut husk maturity and industrial optimization influence particle size and uniformity. The larger diameter and broader distribution observed in the y-CNCs reflect the less organized cellulose structure in young husks, which facilitates less selective hydrolysis. The substantially higher aspect ratio of the c-CNCs (41), resulting from its unique combination of smallest diameter (11 nm) and the longest length (450 nm), contributes to its superior reinforcement performance in polymer matrices through more effective stress transfer and load distribution [13,30].

### 3.3. FTIR Spectroscopy Analysis

**Figure 4:**
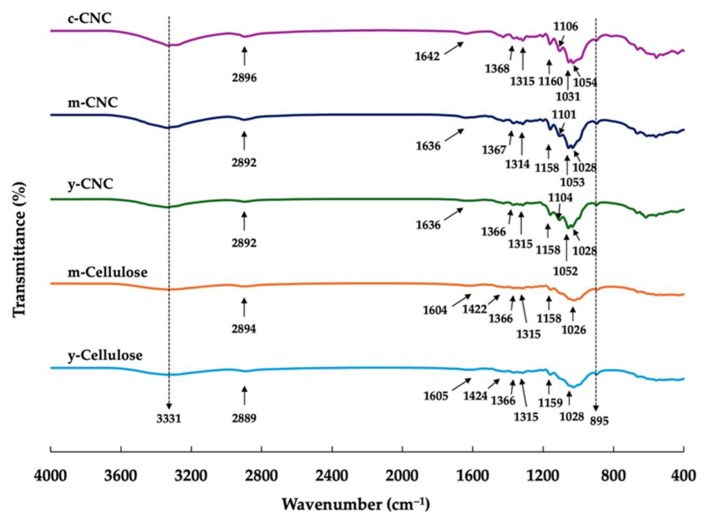
FTIR spectra of cellulose and cellulose nanocrystals (CNCs) from young coconut husk (y-CNC) and mature coconut husk (m-CNC) in comparison with commercial CNC (c-CNC).

FTIR spectroscopy was utilized to examine the chemical composition and to identify functional groups in cellulose and CNCs. Figure 4 presents the FTIR spectra of the cellulose samples (y-cellulose and m-cellulose), the coconut husk-derived CNCs (y-CNCs and m-CNCs), and the c-CNCs. All materials displayed typical cellulose absorption patterns, consistent with spectral characteristics previously documented for CNCs extracted from diverse lignocellulosic sources such as bamboo [12], rice straw [12], *C. sativus* peels [23], poplar wood [24], and sugarcane bagasse [31]. Both cellulose and CNC samples showed a broad band spanning 3200–3500 cm^−1^, corresponding to O–H stretching of hydroxyl groups within cellulose molecules [32]. The breadth of this band indicates the extensive hydrogen bonding interactions inherent to cellulose architecture. An absorption peak near 2892 cm^−1^, representing C–H stretching in methylene and methine moieties of the cellulose chain, appeared in all analyzed samples. Transmittance bands between 1636 and 1642 cm^−1^, present across all spectra, arise from H–O–H bending of adsorbed moisture, reflecting the hydrophilic character of cellulosic materials [33,34]. The band at 1367 cm^−1^ signifies C–H and C–O bending within polysaccharide ring structures, whereas the strong absorption at 1054 cm^−1^ relates to C–O–C stretching of the pyranose ring (antisymmetric in-phase mode), a signature feature of glycosidic bonds in cellulose [32]. Furthermore, the 895 cm^−1^ peak indicates β(1→4)-glycosidic linkages connecting glucose monomers, serving as a definitive cellulose marker. A critical observation was the presence of a peak at 1602 cm^−1^, attributed to aromatic C=C vibrations characteristic of lignin [32], exclusively in y-cellulose and m-cellulose, while being completely absent in the y-CNCs, m-CNCs, and c-CNCs. This absence confirms efficient lignin elimination during acid hydrolysis. The loss of lignin-associated signals in CNC spectra confirms successful purification and extraction of nanocrystals from the lignocellulosic feedstock. The spectral profiles of the y-CNCs and m-CNCs closely resembled that of the c-CNCs, indicating that coconut husk-derived CNCs possess chemical structures and functional groups equivalent to commercial products. In summary, all CNC materials demonstrated identical characteristic cellulose functionalities with no detectable lignin or hemicellulose remnants, validating effective CNC isolation via acid hydrolysis.

### 3.4. XRD Analysis

**Figure 5:**
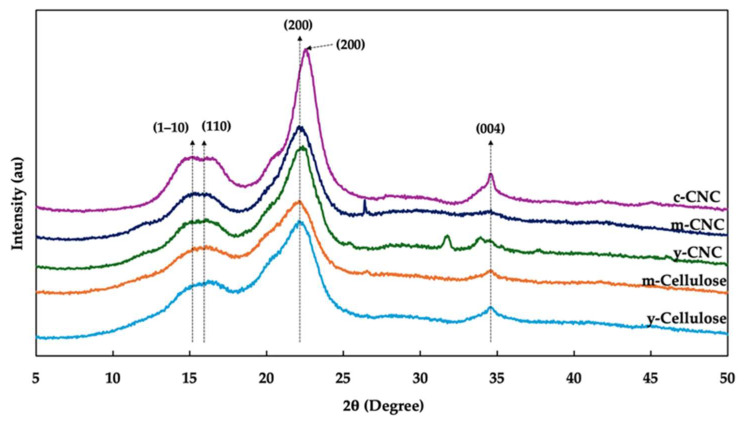
XRD pattern of cellulose and cellulose nanocrystals (CNCs) from young coconut husk (y-CNC) and mature coconut husk (m-CNC) in comparison with commercial CNC (c-CNC).

XRD analysis was employed to investigate the crystalline structure of the y-CNCs and m-CNCs in comparison to the c-CNCs. As shown in Figure 5, all samples exhibited characteristic diffraction patterns of cellulose I crystal structure. The y-CNCs and m-CNCs showed main diffraction peaks at 2θ of approximately 15.3°, 22.2°, and 34.5°, corresponding to the (1–10), (200), and (004) crystallographic planes, respectively [12,35]. Notably, the c-CNCs displayed a more complex diffraction pattern with peaks at 15.1°, 16.2°, 20.7°, 22.5°, and 34.5°, assigned to the (1–10), (110), (012), (200), and (004) planes, respectively. The distinct peak separation at 15.1° and 16.2° in the c-CNCs, corresponding to the (1–10) and (110) planes, respectively, indicates higher crystalline order and better-defined crystal structure compared to coconut husk-derived CNCs, where these peaks appear as a single overlapping peak at 15.3°. Additionally, the appearance of the (012) reflection at 20.7° in the c-CNCs, which is absent or negligible in the y-CNCs and m-CNCs, is characteristic of highly crystalline cellulose I with larger and more perfect crystallites [17]. These differences in peak resolution and the presence of additional minor reflections visually confirm the higher crystallinity of the c-CNCs.

The calculated CrI values are presented in Table 1. The y-CNCs and m-CNCs had CrI values of 61.56% and 63.98%, respectively, while the c-CNCs exhibited the highest CrI value (76.59%). This result indicates that both coconut husk-derived CNCs possess well-organized cellulose structures with pronounced crystalline regions, though with lower crystalline perfection compared to commercial material. Between the two coconut husk-derived CNCs, the y-CNCs showed a lower CrI value than the m-CNCs. This difference can be attributed to maturity-related changes in the chemical composition of coconut husk. Young and mature coconut husks differ in their cellulose content, crystallinity, and the proportion of non-cellulosic components (hemicellulose and lignin) [10,36]. The higher CrI value of the m-CNCs is consistent with the higher cellulose yield obtained from mature coconut husk, as more organized and crystalline cellulose is preferentially retained during acid hydrolysis. The lower crystallinity and broader peak profiles observed in the coconut husk-derived CNCs compared to the c-CNCs can be attributed to several factors. The crystallinity of CNCs depends on their cellulose source and pretreatment methods [37]. Differences in the cellulose content, the extent of amorphous component removal, and the original cellulose crystallite size in the native fiber are among the primary reasons for variations in cellulose crystallinity among various plant sources [38]. Additionally, the final particle size distribution, morphology, and crystalline perfection of CNCs depend on the structure and morphology of the original plant source, acid concentration, temperature, hydrolysis duration, procedure, and any additional mechanical treatment applied [33]. Commercial CNCs, typically derived from wood pulp under highly optimized industrial conditions with standardized hydrolysis parameters, yields materials with superior crystallinity and larger, more perfect crystallites [17]. This observation aligns with Smyth et al. [39], who reported that CNCs isolated from corn (agricultural residue) had lower CrI values than commercial CNCs, concluding that the hydrolysis procedure for CNCs derived from agricultural waste has not been fully optimized for maximum crystallinity. Rosa et al. [37] reported similar findings for CNCs from unripe coconut husks, further supporting the influence of biomass maturity and processing conditions on crystalline properties. However, the CrI values observed in this study (61.56–63.98%) are higher than those reported for CNCs isolated from mulberry bark (60%) and sugarcane bagasse (53%) [12] and comparable to CNCs from other lignocellulosic agricultural residues [40,41,42]. These CrI values are sufficient to provide effective reinforcement within biopolymer matrices. The well-defined cellulose I crystal structure and moderate-to-high crystallinity of the coconut husk-derived CNCs, particularly the m-CNCs, make them suitable as sustainable reinforcing agents for biopolymer composite applications.

### 3.5. Particle Size by DLS

DLS was used to measure the hydrodynamic diameter of CNCs in aqueous suspension [29]. Although DLS is based on spherical particle approximation and may overestimate dimensions for rod-shaped nanoparticles [38], it provides reliable comparative assessment of colloidal behavior and aggregation state among different CNC samples. As shown in Table 1, the average particle sizes of the y-CNCs, m-CNCs, and c-CNCs were 435, 199, and 153 nm, respectively. These size differences can be attributed to variations in fiber morphology, cell wall architecture, and susceptibility to acid hydrolysis associated with biomass maturity and botanical origin [9,19]. Young coconut husk fibers possess a higher proportion of amorphous regions and longer, less compacted cellulose microfibrils, which reduce the efficiency of acid hydrolysis, leading to larger CNC aggregates. In contrast, mature coconut husk exhibits increased secondary cell wall development and structural compaction, facilitating more effective hydrolytic cleavage and resulting in smaller, more individualized particles [18,20]. Commercial CNCs, typically produced under optimized and standardized hydrolysis conditions from wood-based cellulose, exhibits the smallest and most uniform particle size distribution [9,19]. These findings are further corroborated by the TEM observations (Figure 3), which revealed longer nanocellulose rods and a greater tendency for loose aggregation in the y-CNCs compared to the m-CNCs. It should be noted that DLS measures the hydrodynamic diameter, which includes the solvation layer and aggregate structures, while TEM visualizes individual particle dimensions; this difference explains why DLS values are typically larger than TEM-measured lengths. Similar maturity-dependent trends in CNC particle size have been reported for other biomass sources. Saallah et al. [29] reported that CNCs derived from unripe banana peel (328 nm) exhibited a larger average particle size than those from ripe banana peel (259 nm). Similarly, Li et al. [18] found that CNCs derived from softwood (523 nm) were larger than those from hardwood (276 nm), concluding that hardwood consists of shorter fibers that are more susceptible to acid hydrolysis. The smaller and more uniform particle size of the m-CNCs has important practical implications for its application as a reinforcing agent in polymer composites. Smaller nanoparticles typically exhibit better dispersion in polymer matrices, thereby enhancing mechanical properties more effectively.

### 3.6. Zeta Potential Analysis of the CNC Suspensions

The stability of the suspension of nanocellulose was assessed based on the electrostatic repulsive forces between the particles. In general, a colloidal suspension with a zeta potential of less than −30 mV or greater than 30 mV is considered stable [43]. In this study, the coconut husk-derived CNCs obtained from acid hydrolysis exhibited a negative zeta potential of ≤−40 mV, which confirms their colloidal stability. Specifically, the y-CNCs and m-CNCs had a zeta potential of −40.3 and −46.8 mV, respectively, while the c-CNCs had a zeta potential of −55.9 mV (Table 1). The dispersion stability of CNCs is due to the electrostatic repulsion between the negative sulfate groups (−OSO_3_^−^) on the surface of the cellulose [44], which are derived from esterification reaction of the hydroxyl groups present on the cellulose surface [27]. The c-CNCs displayed the most negative zeta potential, indicating excellent colloidal stability. The m-CNCs showed good stability, while the y-CNCs exhibited the least negative zeta potential. Excellent dispersibility of CNCs, indicated by a more negative zeta potential, is a desirable characteristic for their potential usage as reinforcing fillers in the packaging industry. The least negative zeta potential of the y-CNCs corresponds to the largest average particle size (Table 1). The differences in zeta potential among the coconut husk-derived CNCs may arise from the native structure and chemical composition of the source material, which may affect the accessibility of cellulose hydroxyl groups for sulfation. The presence of residual lignin or hemicellulose on the particle surface could sterically hinder sulfate group incorporation or reduce the effective surface charge density [45,46].

### 3.7. Thermal Stability of Cellulose and CNCs

**Figure 6:**
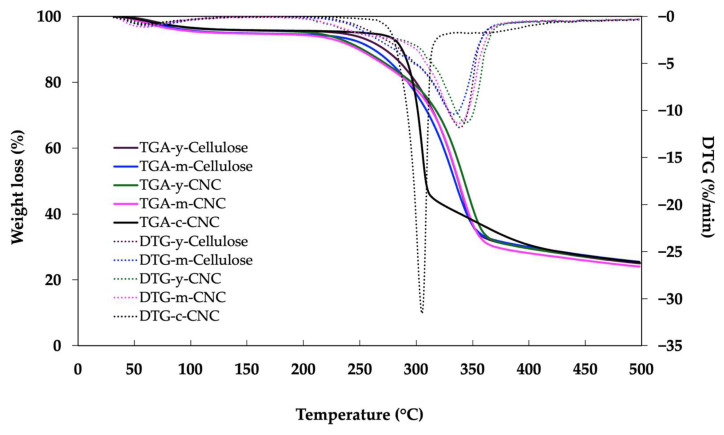
TGA and DTG results of cellulose and cellulose nanocrystals (CNCs) from young coconut husk (y-CNC) and mature coconut husk (m-CNC) in comparison with commercial CNC (c-CNC).

The thermal stability of y-cellulose and m-cellulose and as well as y-CNCs, m-CNCs, and c-CNCs was investigated using TGA and derivative thermogravimetry (DTG) from 25 to 500 °C. Figure 6 illustrates the weight loss curves and their derivatives as a function of temperature. Cellulose samples exhibited two-stage decomposition patterns. The first stage (50–150 °C) showed approximately 5% weight loss attributed to the evaporation of volatile components and absorbed moisture [31]. The second major thermal degradation occurred between 250 and 400 °C with approximately 70% weight loss, corresponding to the pyrolysis of cellulosic material. The degradation onset temperature (T_onset_) was 298 °C for y-cellulose and 293 °C for m-cellulose, indicating good thermal stability of the purified cellulose. Coconut husk-derived CNCs displayed distinct thermal degradation behavior with three stages. The first stage (25–150 °C) involved moisture evaporation and removal of loosely bound water with minimal weight loss. The second stage (150–300 °C) showed weight loss of approximately 12% for the y-CNCs and 9% for the m-CNCs, likely related to the degradation of residual amorphous cellulose, depolymerization of cellulose chains, and decomposition of sulfate ester groups introduced during acid hydrolysis [30]. The third degradation stage (>300 °C) corresponded to the decomposition of crystalline cellulose. In this region, the y-CNCs and m-CNCs exhibited weight loss of 58% and 61%, respectively, while the c-CNCs showed 70% weight loss.

The DTG curves revealed a maximum degradation rate temperature (T_max_) of 344.3 °C for the y-CNCs, 338.2 °C for the m-CNCs, and 305.3 °C for the c-CNCs. The observation that the m-CNCs exhibited a slightly lower T_max_ compared to the y-CNCs despite greater crystallinity can be explained by the influence of sulfate ester groups introduced during acid hydrolysis. The smaller particle size of the m-CNCs (199 nm) compared to the y-CNCs (435 nm; Table 1) results in a significantly higher surface area-to-volume ratio, which leads to a greater density of surface-grafted sulfate groups per unit mass. This is corroborated by the more negative zeta potential for the m-CNCs (−46.8 mV) compared to the y-CNCs (−40 mV; Table 1), indicating a higher sulfate group content. These sulfate groups are thermally unstable and catalyze cellulose degradation through dehydration reactions, thereby reducing the maximum degradation temperature [47,48]. Additionally, the high surface area of CNCs potentially contributes to reduced thermal stability due to increased exposure to heat and more available sites for degradation initiation [49]. The y-CNCs and m-CNCs both demonstrated substantially higher thermal stability than the c-CNCs, which is advantageous for processing and applications requiring elevated temperatures. At 500 °C, the residual char content was approximately 25% for the y-CNCs, m-CNCs, and c-CNCs, indicating similar levels of carbonaceous residue formation. This similarity suggests comparable thermal decomposition pathways despite differences in source materials and processing conditions. Overall, all CNCs demonstrated excellent thermal stability with maximum degradation temperatures above 300 °C, making them suitable for processing conditions typically used in biopolymer composite fabrication and a wide range of industrial applications. The maturity stage of coconut husk (young vs. mature) had a minimal impact on the overall thermal properties, with both sources yielding CNCs with similar decomposition patterns and excellent thermal stability suitable for high-temperature processing applications.

### 3.8. Morphology of the Gelatin-Based Films Reinforced with CNCs

**Figure 7:**
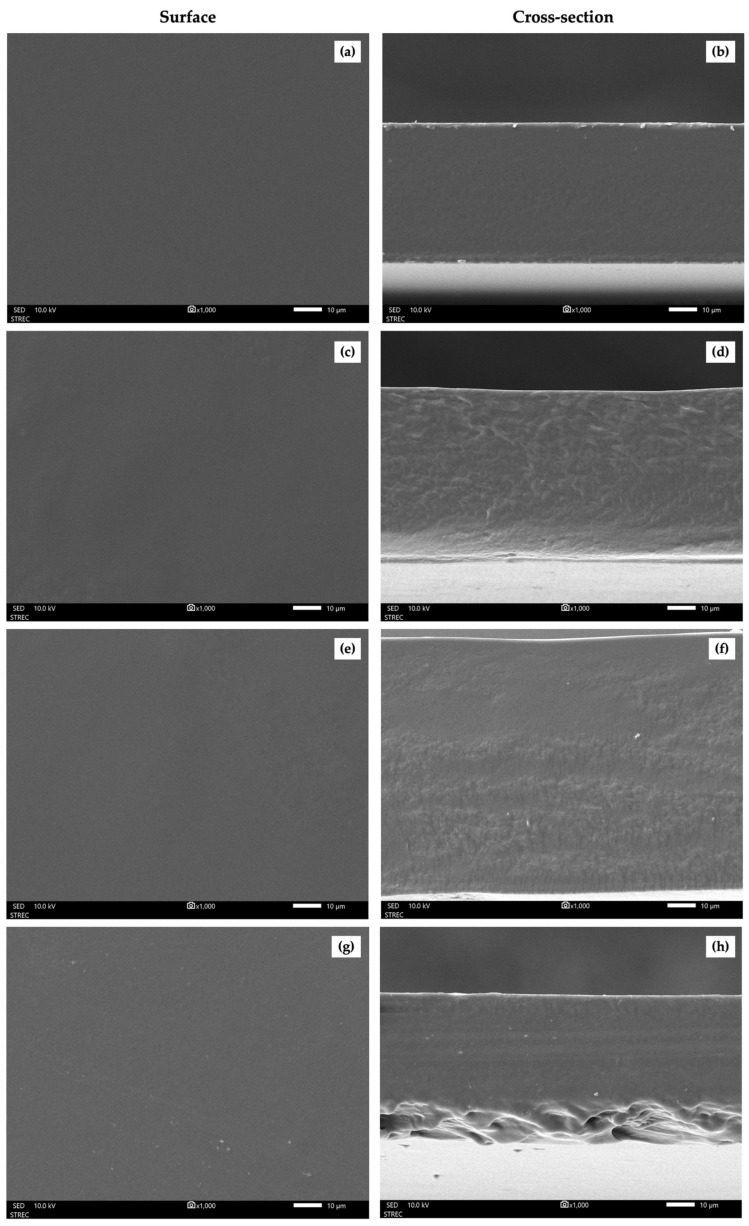
Surface and cross-section (magnification: 1000×) of neat gelatin film (**a**,**b**), G/y-CNC film (**c**,**d**), G/m-CNC film (**e**,**f**), and G/c-CNC film (**g**,**h**). Scale bar: 10 µm for surface and cross-section.

Figure 7 presents the surface and cross-sectional morphology of the G, G/y-CNC, G/m-CNC, and G/c-CNC films based on SEM. The G film exhibited a smooth and homogeneous surface. The cross-sectional image revealed a dense, continuous, and uniform structure without visible cracks, pores, or defects, characteristic of pure gelatin films. The film showed a clear layered structure with smooth interfaces. Upon incorporation of CNCs, all reinforced films maintained a relatively smooth surface morphology, indicating good compatibility between the CNCs and gelatin matrix. However, Ratna et al. [50] found that the incorporation of CNC into the polymer matrix resulted in a rougher surface texture, reflecting an observable microstructural modification. Furthermore, the presence of white dots on the surface can be attributed to the formation of small CNC aggregates as well as the increased cross-sectional density of nanocrystals embedded within the polymer matrix. These morphological observations are consistent with previous studies on CNC-reinforced gelatin films. Tessaro et al. [51] reported that gelatin films reinforced with soybean straw-derived CNCs exhibited smooth surfaces and compact, uniform cross-sectional structures when CNCs were well-dispersed.

There were notable differences in the cross-sectional structures among the reinforced films. The G/y-CNC film showed a rougher, more heterogeneous cross-sectional morphology with visible texture variations throughout the matrix. In contrast, the G/m-CNC film displayed a smoother and more uniform cross-sectional structure, suggesting better CNC dispersion and integration within the gelatin matrix. The G/c-CNC film exhibited some surface roughness with visible small particles, and its cross-section showed a textured structure, indicating the presence of dispersed CNC throughout the matrix. The improved cross-sectional uniformity of the G/m-CNC film compared to the G/y-CNC film can be attributed to the smaller particle size and higher surface charge of the m-CNCs, characteristics that facilitate better dispersion and reduce aggregation [51,52]. Importantly, the G/m-CNC film had a comparable morphology to the G/c-CNC film, with both showing effective CNC incorporation into the gelatin matrix. These morphological observations support the mechanical and barrier property results, where the m-CNCs demonstrated reinforcement performance comparable to the c-CNCs, confirming that mature coconut husk is a viable source for producing effective reinforcing nanomaterials for biopolymer composites.

### 3.9. FTIR of Gelatin-Based Films Reinforced with CNCs

**Figure 8:**
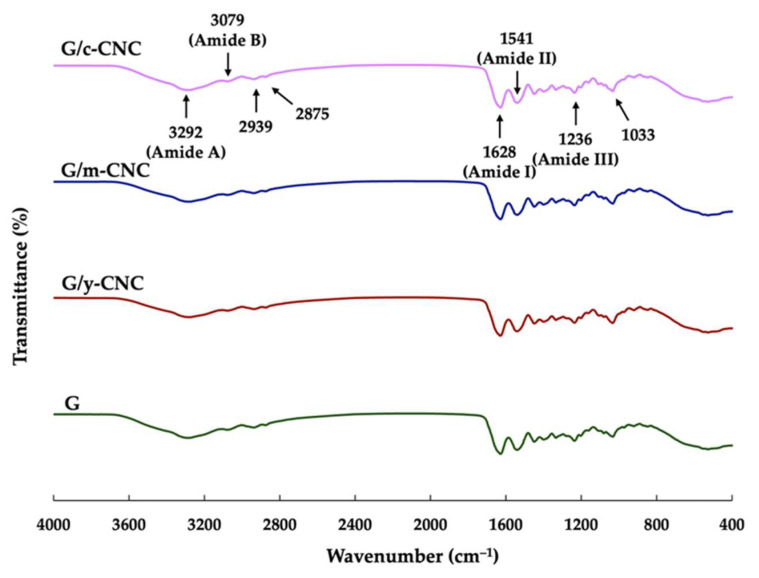
FTIR spectra of the neat gelatin film and gelatin films reinforced with CNCs.

The FTIR spectra of the G, G/y-CNC, G/m-CNC, and G/c-CNC films are presented in Figure 8. All film samples displayed characteristic protein absorption bands with comparable spectral patterns. This finding is consistent with Sun et al. [53], who demonstrated that the addition of CNCs into the polymer matrix did not generate any new characteristic peaks in the FTIR spectrum, suggesting that the interaction between CNCs and the gelatin matrix was primarily governed by physical interactions and hydrogen bonding rather than the formation of new covalent bonds. The spectral region between 700 and 1700 cm^−1^ showed typical amide-I, amide-II, and amide-III absorption bands for all samples. Principal absorption peaks were identified at 1628 cm^−1^ (amide-I band, attributed to C=O stretching vibrations and hydrogen bonding combined with C–N stretching and CCN deformation), 1541 cm^−1^ (amide-II band, corresponding to N–H bending and C–N stretching vibrations), and 1236 cm^−1^ (amide-III band, associated with in-plane C–N and N–H vibrations of bonded amide groups along with CH_2_ wagging vibrations from glycine backbone and proline side chains). The amide-A band, typically observed at 3292 cm^−1^ and representing N–H stretching coupled with inter- or intramolecular hydrogen bonding [54,55], underwent a notable shift upon nanocellulose incorporation. Specifically, this band shifted to a lower wavenumber (3273 cm^−1^) in CNC-reinforced films, coinciding with the enhanced TS observed in these nanocomposite films (Table 2). This spectral shift indicates hydrogen bond formation between nanocellulose and gelatin molecules. Comparable observations have been reported by Onyeaka et al. [56] and Padilla et al. [57], who attributed such changes to hydrogen bonding interactions between gelatin molecules and the crystalline structure of nanocellulose, with concurrent strengthening of inter- and intramolecular O–H and N–H hydrogen bonds within the gelatin network [56]. Sun et al. [53] observed notable shifts in peak positions and variations in peak intensity, particularly within the N–H/O–H stretching region, which were indicative of changes in the hydrogen bonding environment upon CNC incorporation. Specifically, the N–H/O–H absorption band in the CNC-modified films shifted to a lower wavenumber, suggesting the establishment of hydrogen bonding interactions between CNCs and the gelatin matrix. Additional absorption bands were observed at 3079 cm^−1^ (amide-B band, representing asymmetric CH stretching vibrations of carbon–carbon double bonds coupled with NH stretching) [54], while peaks at approximately 2875 cm^−1^ and 2939 cm^−1^ corresponded to symmetric and asymmetric stretching vibrations of aliphatic C–H groups in CH_2_ and CH_3_, respectively. These spectral characteristics confirm successful incorporation of the y-CNCs and m-CNCs into the gelatin matrix through strong hydrogen bonding interactions with the polymer chains, demonstrating the potential of coconut husk-derived CNCs as viable sustainable alternatives to commercial nanocellulose.

### 3.10. Mechanical Properties of Gelatin-Based Films Reinforced with CNCs

The mechanical properties of films are critical indicators of their ability to withstand applied forces and are commonly assessed through TS and EAB. The mechanical properties of gelatin-based films reinforced with y-CNCs, m-CNCs, and c-CNCs are presented in Table 2. The incorporation of CNCs significantly increased the thickness from 0.052 mm for the G film to 0.062–0.063 mm for the nanocomposite films (*p* < 0.05). Theoretically, the addition of nanocellulose would increase the total solid content in the FFS, leading to the formation of thicker films. The addition of CNCs dramatically improved the TS of the gelatin films. The TS increased from 15.63 MPa (the G film) to 21.78 MPa for the G/y-CNC film, 24.25 MPa for the G/m-CNC film, and 24.93 MPa for the G/c-CNC film, representing an increase of 28%, 36%, and 37%, respectively (*p* < 0.05). The superior reinforcement performance of the m-CNCs compared to the y-CNCs, with performance approaching that of the c-CNCs, can be attributed to its smaller particle size, higher crystallinity, and better dispersion within the gelatin matrix, as evidenced by morphological analysis (Figure 7). This enhancement is further supported by strong hydrogen bonding between gelatin and CNCs, as confirmed by FTIR spectroscopy, combined with the rod-like nanocrystals acting as physical reinforcing agents that effectively transfer stress throughout the polymer matrix. Onyeaka et al. [56] reported similar results: They found that the addition of CNCs to gelatin films increased the TS but did not change the EAB when compared with the control gelatin film (without the addition of CNCs). They also concluded that the similarity of the CNC structure to the collagen fiber structure, together with intermolecular hydrogen bonding, collectively withstands stress during the stretching process, consequently resulting in no significant alteration in EAB of the gelatin film [56].

Regarding film flexibility, the coconut husk-derived CNCs did not significantly affect EAB, with a value of 57.32% for the G/y-CNC film and 55.53% for the G/m-CNC film, comparable to the G film (57.83%; *p* > 0.05). Interestingly, the G/c-CNC film showed a significant increase in flexibility, with EAB increasing to 66.85% compared to the G film (*p* < 0.05). This selective response can be attributed to differences in particle size, surface charge, and dispersion quality among the CNC sources, which may have facilitated improved chain mobility and flexibility within the gelatin matrix [58]. Furthermore, Alves et al. [59] explained that nano-sized CNCs with a high surface area can form strong polar interactions with plasticizers such as glycerol, enhancing the dispersion of both components and creating a more ductile composite matrix. Sun et al. [53] recently documented a similar trend, with a 19.82% increase in EAB after the incorporation of CNCs into gelatin films. The authors attributed this enhancement primarily to the strong hydrogen bonding interactions established between CNCs and gelatin molecules, which contributed to improved flexibility of the composite film. Furthermore, the observed increases in EAB have been reported in other biopolymer-based composited films. For example, Ferraz et al. [60] and Thipchai et al. [12] documented an improvement in EAB in carboxymethyl cellulose films containing eucalyptus-derived CNCs and cassava starch films reinforced with bamboo-derived CNCs, respectively. Overall, these results demonstrated that coconut husk-derived CNCs, particularly the m-CNCs, effectively enhanced the mechanical properties of gelatin films with performance comparable to the c-CNCs, confirming its potential as a sustainable and effective reinforcing agent for biopolymer composite applications.

### 3.11. WVP of Gelatin-Based Films Reinforced with CNCs

WVP is a critical parameter for packaging applications, where controlled moisture transfer is essential for food preservation. The WVP values of films ranged from 2.43 to 2.65 × 10^−10^ g m m^−2^ s^−1^ Pa^−1^ (Table 2). The incorporation of m-CNCs resulted in a WVP of 2.43 × 10^−10^ g m m^−2^ s^−1^ Pa^−1^, representing an 8% reduction compared to G film (2.65 × 10^−10^ g m m^−2^ s^−1^ Pa^−1^; *p* < 0.05). The reduction in WVP with m-CNC incorporation can be attributed to the formation of a more compact film structure through strong hydrogen bonding between gelatin and well-dispersed CNCs, which increases tortuosity and thus creates a more tortuous pathway for water vapor diffusion through the matrix [56]. The better dispersion of the m-CNCs compared to the y-CNCs, as evidenced by the morphological analysis, further contributes to its more effective barrier properties. Ratna et al. [50] demonstrated that the incorporation of CNCs into gelatin/carboxymethyl cellulose composite films led to a reduction in WVP. The authors attributed this change to the ability of CNCs to fill interfacial pores and to connect polymer chains, thereby decreasing both the number and size of pores within the film matrix. Additionally, CNCs may act as physical barriers that compel water molecules to travel along longer, more tortuous diffusion pathways through the film, a phenomenon commonly referred to as the tortuosity effect [50]. Collectively, these results demonstrate that the m-CNCs effectively reduced the WVP of gelatin films, with performance comparable to the c-CNCs, further confirming its suitability as a sustainable and effective reinforcing agent for food packaging applications.

### 3.12. WCA of Gelatin-Based Films Reinforced with CNCs

WCA measurements evaluate surface wettability, where angles exceeding 90° signify hydrophobic characteristics while angles below 90° indicate hydrophilic behavior. As shown in Table 2, all films exhibited hydrophobic characteristics, with WCAs ranging from 115.7° to 127.5°. The G film showed a WCA of 125.6°, indicating good water resistance. The incorporation of CNCs generally maintained this hydrophobic character, with no significant differences observed for most formulations (*p* > 0.05). The G/m-CNC film showed a WCA of 125.3°, comparable to the G/c-CNC film (127.5°), indicating that the m-CNCs maintain the water-resistant properties of gelatin films at levels similar to the c-CNCs. The G/y-CNC film exhibited a slightly lower WCA (115.7°), which may be attributed to surface roughness created by the larger y-CNC particles protruding from the film surface, as observed in the cross-sectional SEM images (Figure 7). Tessaro et al. [51] reported similar reductions in the WCA with the addition of CNCs to gelatin films reinforced with soybean straw nanocellulose, correlating with increased film thickness and surface roughness. Overall, the high WCAs of all films (>115°) indicate low surface wettability and good moisture resistance, making them suitable for packaging applications requiring water barrier properties, particularly for dry food products. The comparable contact angles of the G/m-CNC and G/C-CNC films further confirm that mature coconut husk is a viable source for producing CNCs with properties similar to commercial alternatives.

### 3.13. UV–Vis Barrier Properties of Gelatin-Based Films Reinforced with CNCs

**Figure 9:**
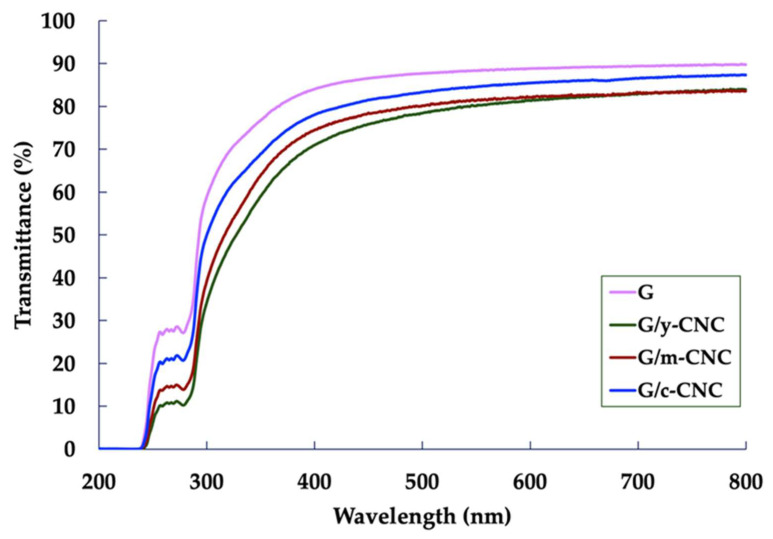
Ultraviolet and visible light barrier properties of the neat gelatin film and the gelatin films reinforced with CNCs.

UV–Vis barrier properties are crucial for food packaging films, as they inhibit lipid oxidation and nutritional degradation caused by light exposure. The light transmission spectra of the G, G/y-CNC, G/m-CNC, and G/c-CNC films across wavelengths of 200–800 nm are presented in Figure 9. The UV light transmission (200–280 nm) and the visible light transmission (350–800 nm) of the G/y-CNC, G/m-CNC, and G/c-CNC films ranged from 0.075% to 21.26% and from 59.06% to 87.38%, respectively, while the transmission for the G film was 0.12–27.75% and 76.80–89.76%, respectively. Lower light transmission indicates better barrier properties. The G film exhibited the highest light transmission across all wavelengths, indicating poor light barrier properties. The incorporation of CNCs reduced light transmission across both UV and visible light regions, with all CNC-reinforced films demonstrating improved barrier performance compared to the G film.

Notably, the G/y-CNC film showed the lowest light transmission, providing the strongest barrier effect among all films tested. This was followed by the G/m-CNC film, which also demonstrated substantial reduction in light transmission compared to the G film. The G/c-CNC film showed moderate improvement. The enhanced light barrier properties of the y-CNCs and m-CNCs can be attributed to effective light scattering by dispersed CNC nanoparticles within the gelatin matrix. This enhanced light blocking is attributed to increased light scattering and absorption by the CNC nanoparticles, which effectively prevents light penetration through the film matrix [61,62]. Similar improvements in light barrier properties have been documented for gelatin films reinforced with soybean straw-derived CNCs [60] and chitosan films with CNCs [62]. These results demonstrate that coconut husk-derived CNCs, particularly the y-CNCs and m-CNCs examined in this paper, effectively enhance the UV and visible light barrier properties of gelatin films, with performance superior to the c-CNCs. This superior light barrier property, combined with the excellent mechanical and barrier properties of the m-CNCs, confirms that coconut husk is an exceptional source for producing high-quality reinforcing nanomaterials for food packaging applications requiring protection from light-induced degradation.

## 4. Conclusions

This study successfully isolated CNCs from young and mature coconut husks, abundant agricultural waste from Thailand’s coconut industry, via acid hydrolysis. FESEM and TEM analyses confirmed that all isolated CNCs exhibited characteristic rod-like morphologies. Notably, the maturity stage of the coconut husk significantly influenced CNC properties: The m-CNCs exhibited higher crystallinity, superior colloidal stability, and a smaller particle size compared to the y-CNCs. All CNCs demonstrated excellent thermal stability above 300 °C. Between the coconut husk-derived CNCs, the m-CNCs showed the most promising results, displaying performance comparable to the c-CNCs. These findings indicate that mature coconut husk can be recognized as a renewable material for producing cellulosic reinforcing materials. However, reinforcing materials can also be prepared from young coconut husk, though further studies are needed to determine the optimal extraction conditions. These CNCs acted as effective reinforcing agents in biopolymer composites, enhancing both mechanical strength and water vapor barrier properties of the gelatin-based films by 28–37% and 8%, respectively. These improvements demonstrate the effectiveness of coconut husk-derived CNCs as sustainable reinforcing agents for biopolymer composites. The findings indicated that mature coconut husk represents one of the most promising renewable sources for producing high-quality CNCs for packaging applications. While CNCs can also be prepared from young coconut husk, further optimization of extraction conditions is needed to improve their properties.

## Figures and Tables

**Table 1:** Specification of cellulose nanocrystals from young (y-CNC) and mature (m-CNC) coconut husk in comparison with commercial cellulose nanocrystals (c-CNC).

Samples	Crystallinity Index (%)	Average Particle Size (nm)	Zeta Potential (mV)
y-CNC	61.56 ± 0.81 ^b^	435 ± 2.14 ^a^	−40.3 ± 0.06 ^a^
m-CNC	63.98 ± 1.73 ^b^	199 ± 2.04 ^b^	−46.8 ± 0.59 ^b^
c-CNC	76.59 ± 1.95 ^a^	153 ± 1.72 ^c^	−55.9 ± 0.45 ^c^

Values are given as mean ± SD (*n* = 3). Different superscripts in each column are significantly different (*p* < 0.05).

**Table 2:** Mechanical properties, water vapor permeability (WVP), and contact angle of the gelatin-based films reinforced with nanocellulose.

Films	TS (MPa)	EAB (%)	WVP (×10^−10^ g m m^−2^ s^−1^ Pa^−1^)	Contact Angle (Degree)
G	15.63 ± 0.62 ^c^	57.83 ± 6.99 ^b^	2.65 ± 0.02 ^a^	125.61 ± 0.75 ^a^
G/y-CNC	21.78 ± 1.07 ^b^	57.32 ± 6.11 ^b^	2.57 ± 0.13 ^a^	115.71 ± 2.86 ^b^
G/m-CNC	24.25 ± 1.91 ^a^	55.53 ± 2.78 ^b^	2.43 ± 0.02 ^b^	125.32 ± 0.96 ^a^
G/c-CNC	24.93 ± 1.81 ^a^	66.85 ± 5.33 ^a^	2.52 ± 0.02 ^ab^	127.50 ± 1.07 ^a^

Values are given as mean ± SD, *n* = 5 for TS and EAB determinations and *n* = 3 for WVP and contact angle determinations. Different superscripts in each column are significantly different (*p* < 0.05).

## Data Availability

The original contributions presented in this study are included in the article. Further inquiries can be directed to the corresponding author.

## Abbreviations

The following abbreviations are used in this manuscript:

CNCs	Cellulose nanocrystals
y-CNC	Young coconut husk cellulose nanocrystals
m-CNC	Mature coconut husk cellulose nanocrystals
c-CNC	Commercial cellulose nanocrystals
G	Gelatin
TS	Tensile strength
EAB	Elongation at break
WVP	Water vapor permeability
